# Undernutrition and Associated Factors among Adolescent Girls in Damot Sore District, Southern Ethiopia

**DOI:** 10.1155/2020/5083140

**Published:** 2020-06-25

**Authors:** Degu Demissie Gagebo, Amene Abebe Kerbo, Thilagavathi Thangavel

**Affiliations:** ^1^Wolaita Sodo University, College of Medicine and Health Science, Department of Human Nutrition, Wolaita Sodo, Ethiopia; ^2^Wolaita Sodo University, College of Medicine and Health Science, Department of Public Health, Wolaita Sodo, Ethiopia

## Abstract

**Background:**

Adolescent girls were given little health and nutrition attention. Focusing on adolescent girls' nutrition prior to conception is one way to break the intergenerational cycle of malnutrition. Therefore, the aim of this study was to assess the prevalence of undernutrition and associated factors among adolescent girls in rural Damot Sore District, Southern Ethiopia.

**Methods:**

A community-based cross-sectional study was conducted from February to March 2017. Multistage sampling technique was used to select 729 adolescent girls. Structured interviewer-administered questionnaire was used to collect information on different variables. Weight and height were measured by using a well-calibrated digital Seca scale and portable stadiometer by trained data collectors. Height-for-age (HFA) and body mass index-for-age (BMIFA) *z*-scores were calculated using WHO AnthroPlus software as indicators of stunting and thinness, respectively. Wealth index was generated by using principal component analysis (PCA), and based on the results, household wealth index/status was converted into tertiles and categorized as higher/rich, medium, and lower/poor. Descriptive statistics, bivariable, and multivariable logistic regression analysis were done. Strength of association of variables was presented by odds ratio along with its 95% CI.

**Results:**

The prevalence of stunting and thinness among adolescent girls was 29.6% (95% CI = 26.6%, 32.8%) and 19.5% (95% CI = 16.7%, 22.3%), respectively. Being in older adolescence (AOR = 2.06, 95% CI = 1.08, 3.92), mother occupation (farmer and government employee) ((AOR = 2.38, 95% CI = 1.31, 4.33) and (AOR = 3.05, 95% CI = 1.35, 6.92)), mother education (secondary and above) ((AOR = 0.53, 95% CI = 0.28, 0.98) and (AOR = 0.25, 95% CI = 0.09, 0.69)), and household wealth index (poor) (AOR = 1.94, 95% CI = 1.29, 2.92) were significantly associated with stunting. Father education (primary and secondary) ((AOR = 0.48, 95% CI = 0.31, 0.77) and (AOR = 0.45, 95% CI = 0.26, 0.78)), mother education (primary) (AOR = 0.56, 95% CI = 0.37, 0.87), and meal frequency (<2/day) (AOR = 1.87, 95% CI = 1.12, 3.13) were significantly associated with thinness.

**Conclusion:**

The prevalence of stunting and thinness among adolescent girls was moderate, when compared to the prevalence reported in Sub-Saharan Africa. However, it was a major public health problem, when compared to the national nutrition baseline survey reports in Ethiopia. Parental education was a significant predictor of both stunting and thinness among adolescent girls. Thus, initiation of routine screening, promotion of education, and implementation of evidence based community nutrition programmes required to be improved.

## 1. Background

According to Ahmed et al., 2009, as cited in Yoseph et al., 2014, adolescence is a decisive period of development because it represents the transition between life as a child and life as an adult covering the ages between 10 and 19 years. During this crucial period, dietary patterns have vital impact on lifetime nutritional status and health [1]. As to the WHO, 2005, as cited in Teji et al., 2015, adolescents make up roughly 20% of the total world population and remarkably 84% of them are in developing countries [2]. In Ethiopia, children and adolescents constitute about 48% of Ethiopian population and of this about 13% are girls according to mini Ethiopian demographic and health survey report of 2014 [3].

Adolescence is the second most critical period of physical growth in life cycle after first year of infancy. It is the most important period of life where growth and development are accompanied by numerous physical, physiological, behavioural, and social changes [4–6]. It is the future generation of any country and meeting their nutritional needs is critical for the wellbeing of a society [7].

Inadequate diet has contributed a lot to the poor nutritional status of adolescents. It is one of the most common causes of morbidity among adolescents throughout the world [1, 8]. Adolescents are facing a series of serious nutritional challenges, which would affect their health at adult age. Hence, the prevalence of undernutrition, particularly, among adolescents is an alarming global problem and affecting about one-third of the world population [7, 9].

According to Rosen, 2004, as cited in Weres et al., 2015, in Sub-Saharan Africa, the prevalence of adolescence undernutrition was 15% to 58%, which is more highly relative to countries of other regions of Africa [4]. A study conducted in eleven developing countries shows that the prevalence of stunting among Kenyan adolescent school girls was 12.1% and among the Nigerian adolescents was 67.3% of boys; 57.8% of girls were stunted and 64.2% of rural Tanzanian adolescents were stunted [10, 11].

In the rural Ethiopia, in contrast to boys, girls face intrahousehold gender discrimination and are treated unfairly in terms of food allocation, opportunity during puberty, and work burdens, which leads to more nutritional problems among adolescent girls. Moreover, their restriction in freedom has compounded prevalence of the problem in girls to larger extent [12–14]. National nutrition baseline survey conducted in Ethiopia reported that girls in rural areas more likely to be stunted with the prevalence of 23% and 14% of adolescent girls had a low body mass index for age (thin) [15].

Girls who are stunted and later become pregnant are likely to give birth to small-for-gestational age (low birth weight) children. Undernourished adolescent girls are more likely to have undernourished mothers and they are more likely to have been undernourished in early childhood and more likely give birth to undernourished offspring in the future, thus transmitting undernutrition to the future generation [16–19].

Short stature resulting from chronic undernutrition is associated with reduced lean body mass and deficiencies in muscular strength and working capacity. Furthermore, malnourished adolescents are suboptimally productive during adulthood, which leads to reduced economic potential and perpetuates the cycle of poverty. Hence, improving adolescent girls' nutrition and health prior to conception is one way to break the intergenerational cycle of malnutrition [19–22].

Most of the extant literatures indicate that much has been known regarding the nutritional status of in-school and pregnant adolescent girls. However, those who were not attending schools and nonpregnant adolescent girls were not given adequate attention they deserve. Moreover, adolescent girl's nutritional status was not included in the national health and nutrition surveys, which is indicative of lack or low interest of policy makers regarding the issue [23–25]. This approach ignores the fact that many health problems later in life can be improved by adapting nutrition intervention and healthy life style during adolescence [5, 26]. Therefore, this study aimed to fill such a gap in assessing the prevalence of undernutrition and associated factors among adolescent girls in rural Damot Sore District, Southern Ethiopia.

## 2. Materials and Methods

### 2.1. Study Setting, Design, and Period

This study was conducted in Damot Sore Woreda (administrative stage) which is located at 320 km away from Addis Ababa to the south and 17 km away from Sodo town in the western direction. The Woreda has 21 kebeles (the smallest administrative unit in Ethiopia) with different climatic zones and rural urban living residences. Among 22 kebeles, 3 are urban whereas 18 of them are rural. Data were collected exclusively from rural kebeles. According to the Woreda Health Office 2016 report, Damot Sore Woreda has the total population of 128,184 and, of this, the number of households with adolescent girls was estimated to be 19,228 [3, 27]. A community based cross-sectional study design was employed among households with adolescent girls aged 10–19 years. The source population for the current study was adolescent girls residing in the Damot Sore district and all households with adolescent girls aged 10–19 years in selected kebeles of Damot Sore district were the study population. Adolescent girls who were pregnant, critically ill, and physically challenged for anthropometric measurements were excluded from the study. The study was conducted from January to February 2017, giving consideration to food surplus season of the year in Ethiopia.

### 2.2. Sample Size Determination and Sampling Technique

The sample size for the study was calculated using single population proportion formula. The assumptions considered to calculate the sample size include 95% confidence level of significance (*Z*_*α*/2_ = 1.96), margin of error 5%, and design effect of 2, and the prevalence of stunting of 31.5% was taken from a community based cross-sectional study conducted among adolescent girls in the Amhara Region, Northern Ethiopia [28], 10% (66 participants) were nonresponsive, and the final samples size was 729 households with adolescent girls.

To ensure sample size sufficiency, sample size was calculated using the prevalence of stunting (31.5%) [28] which was 729, calculated using prevalence for thinness (21.6%) [2] which was 572, and also the sample size was calculated using the relevant variables which were significant predictors of both stunting and thinness in other related studies. However, the total sample size calculated using all others was less than the one calculated using the prevalence of stunting.

A multistage stage sampling technique was employed. In the first stage, from 18 rural kebeles, seven kebeles were selected by lottery method. According to the size of adolescent girls' population, sample size was distributed to each kebele proportionally. In the second stage, study participants (households with at least one adolescent girl) were selected using systematic sampling technique by preparing sampling interval (*N*/*n* = 11) using the sampling frame obtained from the list of households with adolescent girls in each kebele family folder (family registration book) or community health information system (CHIS). The first household was selected by choosing one random number out of the sampling interval by lottery method and every 11^th^ household was included until the required sample size was achieved. The direction to start at the first household was selected randomly. In cases where there are two or more adolescent girls in the same household, one of them was selected randomly by lottery method. In another case when there is no adolescent girl in the selected household, the adjacent/next household was selected.

### 2.3. Data Collection Procedures and Instruments

For data collection, face-to-face, structured interviewer administered questionnaire and anthropometric measurements were used. The questionnaire consisting of sociodemographic and economic information, food/dietary intake pattern, reproductive health, morbidity status, and information regarding other associated factors that are extracted from different literatures was used as data collection tool. The questionnaire was initially prepared in English and translated to local language (Wolaitigna) and then translated back to English to check the consistency by language experts. A total of 14 diploma holder female nurses who were fluent speakers of the local language were recruited and collected the data, considering prior experience of participation in anthropometric data collection, and two public health officers supervised the data collection. The data collectors were trained on the data collection procedures, the context of specific questions across the questionnaire, and anthropometric measurement procedures. In order to include those adolescent girls in schools and who were absent at the time of the interview, households selected were revisited in the weekends and the required information was collected.

Anthropometric assessment constitutes weight and height measurements. Weight was measured using a well-calibrated, portable digital seca scale to the nearest 0.1 kg. Height was measured using a portable stadiometer, which consisted of an anthropometer with a simple triangular headboard to the nearest 0.1 cm. The participants were measured for weight and height by taking off shoes, heavy clothes, and mobile from the pocket, by standing upright/straight with their head held erect and their shoulder blade, buttocks, back of the head, and heel touching the scale and with knees and arms hanging naturally by the sides, and by standing on the foot mark on scale such that the external auditory meatus and the lower boarder of the eye were in one horizontal plane (Frankfurt plane), respectively, according to the WHO recommendations [29]. WHO AnthroPlus computer program was used to assess nutritional status in terms of stunting and thinness of adolescent girls. The anthropometric measurements were converted into height-for-age *z*-scores and BMI-for-age *z*-scores and compared to the new 2007 WHO reference data for 5–19 years [29, 30]. Stunting and thinness were defined as height-for-age and body mass index-for-age < −2 *z*-scores of the references according to predetermined CDC cut-off points and WHO reference data of 5–19 years' population.

### 2.4. Data Quality Control and Standardization

Questionnaire was first prepared in English and translated to Wolaitigna language by language experts and then translated back to English to maintain its consistency. Both data collectors and supervisors were trained by principal investigator for three days on the objective, relevance of the study, the operation of the weight and height measurement scales data collecting and interviewing approach, how to select adolescents from the household, respondent's right, proper filling of questionnaire, and data recording. The questionnaire was pretested on 5% of the sample size from two kebeles (Demba Zamine and Shakisho) which were not selected for the actual study. Based on the pretest, validity and reliability of the measurement was checked, questions that posed difficulty were revised and edited, and those found to be unclear or confusing were removed.

To assure the accuracy of anthropometric measurement, standardization test was done on 10% (73 participants) before the actual survey and systematized based on the results. To do standardization test, the seventy-three adolescents were selected and height and weight were measured twice by the principal investigator. Then, the two anthropometric data collectors measured the same adolescents twice with some time interval. The anthropometric data then entered into ENA SMART software to see relative Technical Error of Measurement (TEM). The TEM was found to be in acceptable range.

Two different measurements were taken for the height and weight by two different measurement takers for every study subject so that the average of the two was considered for the analysis when two measurements differ by one unit. This would help in reducing the occurrence of measurement errors by single individual measurement to avoid interobserver and intraobserver errors [29]. To improve the quality of data, the data collectors were closely supervised. Completeness, accuracy, and consistency of the collected data were checked on daily basis during data collection by the supervisors and principal investigator. Any filled questionnaire that was a difficult to understand was rejected from the study. The principal investigator was responsible for coordination and supervision of the overall data collection process.

### 2.5. Data Processing and Analysis

Before data entry and cleaning, the data were checked manually for completeness and consistency. Data were coded and entered into EpiData version 3.1 and exported to SPSS version 20 for analysis. Anthropometric data were entered and analysed using AnthroPlus software. Principal component analysis (PCA) was used to generate wealth index. Based on PCA, the results of household wealth status/index were converted into tertiles and categorized into higher/rich, medium, and lower/poor tertiles.

Descriptive statistics using frequency with proportions, mean, standard deviations, and correlation were used to present study results. All continuous variables were checked for normality by using the Kolmogorov–Smirnov test at *p* value >0.05. Age, height, and weight were described using mean and/or median. Bivariate and multivariate logistic regression was done to assess the association between adolescent undernutrition in terms of stunting and thinness. Before inclusion of predictor variables, multicollinearity was also checked among selected variables by using cut-off point of VIF < 10 and tolerance test greater than 0.1. Hosmer and Lemeshow goodness of fit test was used to assess fitness of the model during multivariable analysis and the model was >60%. Strength of association was measured using both crude and adjusted odds ratios along with 95% confidence interval. *p* value less than 5% (0.05) was considered to declare statistical significance of the dependent variable with independent variables.

Flagged cases were considered as indicators of extreme or potentially incorrect *z*-score values. Corrections were made immediately checking any data entry errors. However, all flagged *z*-scores after corrections had been considered for any data entry errors were excluded from the analysis. The cut-off points for HFA and BMIFA to be considered as flagged case were ±6 for HFA and ±5 for BMIFA, respectively. There were totally ten (five in stunting and five in thinness) flagged cases during nutritional data analysis and were excluded from the analysis.

### 2.6. Operational Definition of Terms

  Stunting is height-for-age *z*-scores below minus two standard deviations (−2 SD) from the median of the new WHO reference population [29, 30]
  Thinness is BMI-for-age *z*-scores less than −2 standard deviations from the median of the new WHO 2007 reference population [29, 30]
  Wealth index is two or more assets owned by a household like farm land, farm instruments, livestock ownership, and durable goods (motor cycle, bicycle, mobile phones, radio, chairs, tables, television, watch, jewellery, and housing quality used for floor, wall, and roof); it is the an indicator of household living standard [18, 24, 31, 32]

### 2.7. Ethical Clearance

Ethical clearance was obtained from the Institutional Research Ethics Review Committee of Wolaita Sodo University. Official letter of cooperation was written to Damot Sore Woreda Health Office for permission to conduct the study. After providing the official letter written by Woreda Health Office to respective kebele leaders, detailed discussions regarding the study were made with kebele leaders and verbal permission was obtained. Additionally, permission to conduct the study in the local area was obtained from kebele leaders. All selected participants were informed about the objective of the study and data were collected after getting informed verbal consent from the participants. For respondents whose age was less than 18 years, informed oral consent was obtained from their parents or caregivers and assent was also sought from the girls. For those aged 19 years, informed verbal consent was obtained from the adolescents themselves. To ensure confidentiality of participants' information, participant codes were used; thereby, the names of the participants and any participants' identifiers were not written on the questionnaire. The respondents were assured that they have the right to refuse or decline the study at any time and not to answer the question they do not want to answer and that refusing to participate on the study could not bring any effect on them.

## 3. Results

### 3.1. Sociodemographic Characteristics of Study Participants

A total of 719 adolescent girls were enrolled in the study, making the response rate of 98.6%. Of the study subjects, 435 (60.5%) were younger/early adolescent girls within the age group of 10–14 years and the median age of respondents was 14 years with the Inter Quartile Range of 3 years. As to religion and ethnicity of respondents, more than half, 374 (52.0%), and almost all, 694 (96.5%), of the respondents were protestant in religion and Wolaita in ethnicity, respectively. As to the family of respondents, over half of the respondents, 437 (60.8%), were from large family size (>five members). The occupational status of study participants' family showed that 355 (49.4%) of their fathers and 317 (44.0%) of their mothers were farmers and housewives, respectively. The educational background of their parents revealed that two hundred eighty-two (39.2%) of their fathers and 311 (43.3%) of their mothers attended primary education. Regarding household wealth index level, two hundred forty-three (33.8%) of the respondents were from poor families.

Majority, 487 (67.7%), of the respondents' families had dry pit latrine with slab. Almost all, 704 (97.9%), of the respondents used water from safe source (tap and spring water) for drinking purposes. As to meal frequency of respondents, more than half, 450 (62.6%), and around 24.3% have consumed their regular meal three times a day. Almost all, 701 (97.5%), of the respondents had no history of skipping their regular meal. Majority, 708 (98.5%), of the study participants had little/no household hunger due to shortage of food (Table 1).

### 3.2. Nutritional Status of Adolescent Girls

The median height and weight of the respondents were 150 cm and 42 kg, respectively. The mean ± SD BAZ and HAZ were −0.41 ± 1.0 and −0.81 ± 0.9, respectively. Nutritional status of respondents was determined by using CDC cut-off points and 2007 WHO growth reference classification for BMI-for-age and height-for-age. According to the result, 2 (0.3%) of the subjects were severely thin with BMI-for-age *z*-scores < -3SD while 138 (19.2%) were thin with BMI-for-age *z*-scores < −2 SD. As to stunting, 4 (0.5%) of the respondents were severely stunted with HAZ < −3SD and 209 (29.1%) were stunted with HAZ < −2 SD, respectively.

**Figure 1:**
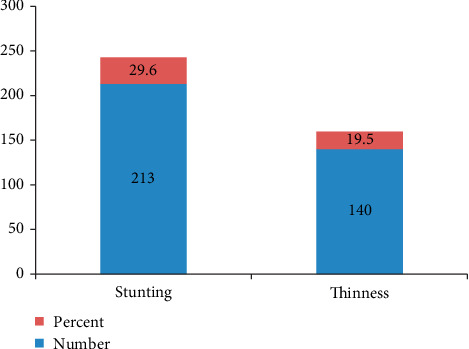
Nutritional status among adolescent girls in Damot Sore Woreda, Southern Ethiopia, 2017.

The overall prevalence of stunting (low height-for-age) and thinness (low body mass index-for-age) among adolescent girls was 29.6% (95% CI; 26.6%–32.8%) and 19.5% (95% CI; 16.7%–22.3%), respectively (Figure 1). Both stunting and thinness were more prevalent among older/late adolescent girls than younger/early adolescent girls: 35.6% vs. 25.7% and 21.8% vs. 17.9%, respectively. This indicates that both stunting and thinness were increasing with the increasing age of girls especially during older adolescence period. Sixty-one of the study participants were both stunted as well as thin. From the stunted girls, 21 (43.8%) had malaria and 13 (56.5%) had diarrhoea whereas, from thin adolescents, 29 (60.4%) had malaria and 21 (91.3%) had diarrhoea.

### 3.3. Factors Associated with Stunting among Adolescent Girls

In a bivariate logistic regression model, variables that were significantly associated (*p* < 0.05) with stunting were being in older adolescent period, mother's occupation, mother's educational status, wealth index, and mother's age. Other variables such as father's occupation, father's education, family size, frequency of meal per day, using soap while washing hands, and girls who started menarche were found to be associated with stunting at *p* value <0.25. The multivariable analysis was performed for variables showed statistically significant association with stunting at bivariate level. However, in multivariable logistic regression, only being in older adolescent age, mother's occupation, mother's educational status, and wealth index were found to be statistically significantly associated with stunting.

The study findings show that older adolescent girls have 2 times higher odds of developing stunting as compared to their counterparts (AOR = 2.06, 95% CI = 1.08, 3.92). Adolescent girls whose mothers' occupation was farmer were 2.4 times (AOR = 2.38, 95% CI = 1.31, 4.33) more likely to be stunted whereas those girls whose mothers' occupation was government employee were 3 times (AOR = 3.05, 95% CI = 1. 35, 6.92) more likely to be stunted as compared to those girls whose mothers' occupation was housewife. Adolescent girls from educated mothers were less likely to be prone to stunting when compared to those from noneducated one. Adolescent girls whose mothers had secondary education have 47.0% (AOR = 0.53, 95% CI = 0.28, 0.98) lower odds of developing stunting while those girls whose mothers attended more than secondary education have 75.0% (AOR = 0.25, 95% CI = 0.09, 0.69) lower odds to develop stunting as compared to mothers who had no formal education. The result also revealed that there was statistically significant association between household wealth index and adolescents' nutritional status. Those adolescent girls whose household wealth tertile was lower/poor were 2 times more likely to be stunted than adolescent girls whose household wealth tertile was higher/rich (AOR = 1.94, 95% CI = 1.29, 2.92) (Table 2).

### 3.4. Factors Associated with Thinness among Adolescent Girls

During bivariable analysis, fathers' education, mothers' education, number of meals being eaten per day, and using soap when washing hands per day were associated with thinness at *p* value <0.05. But variables like girls' age, family size, girls who started menarche, and wealth index were associated with being thin at *p* value <0.25. However, most variables lost their significance in the multivariable model and only fathers' education, mothers' education, and frequency of meal per day were found to be statistically significantly associated with being thin.

Adolescent girls whose fathers' education level was primary were 52.0% (AOR = 0.48, 95% CI = 0.31, 0.77) less likely to be thin while girls whose fathers' education was secondary level were 55.0% (AOR = 0.45, 95% CI = 0.26, 0.78) less likely to be thin as compared to those girls whose fathers had no formal education. Similarly, adolescent girls whose mothers' education was primary level were 44.0% less likely to be thin when compared to those girls whose mothers had no formal education (AOR = 0.56, 95% CI = 0.37, 0.87). Those female adolescents who usually eat two meals and less per day were 1.8 times (AOR = 1.87, 95% CI = 1.12, 3.13) more likely to be thin as compared to those who usually eat three meals per day (Table 3).

## 4. Discussion

This study has attempted to determine the prevalence and factors associated with stunting and thinness among rural adolescent girls. The results of current study indicated that the prevalence of stunting and thinness was 29.6% and 19.5%, respectively. The study findings also revealed that older adolescent girls, mothers' education, mothers' occupation, and wealth index were statistically significantly associated with stunting while fathers' education, mothers' education, and frequency of meal per day were found to be significantly associated with thinness.

The prevalence of stunting, 29.6%, was in line with the study conducted in Amhara Region which was 31.5% and the studies conducted in rural Varanasi, in Kathmandu, in rural Kolar district, and in rural area of central India which were 26.6%, 32%, 32.2%, and 30.4%, respectively [23, 28, 33–35]. However, the prevalence was higher than that of the studies conducted in Babile District, Eastern Ethiopia, in Addis Ababa, Ethiopia, in Adowa town, Northern Ethiopia, in Adama city, central Ethiopia, and in urban areas of northern Tigray which was 15%, 7.2%, 12.2%, 15.6%, and 21.2%, respectively [1, 2, 19, 22, 36].

The variation in the results of different studies might be because this study represents the population exclusively from the rural setup, whereas other studies represented the population from the urban setup as well in which there is an improvement in sociodemographic and socioeconomic condition in urban setting. Another possible explanation might be due to the study setting in which those studies were conducted in institution/school based while the current study was conducted in the community setting. The other possible explanation for the high prevalence could be attributed to the nature of stunting, which indicates long term cumulative inadequacy of nutrition that remained uninterrupted throughout the adolescent life and suggests nutritional deprivation in early childhood. Hence, adolescents were more affected by stunting despite improving conditions over time nowadays.

The prevalence of thinness, 19.5%, also goes in line with that of a study conducted in Adwa, a study conducted in Wukro, Northern Ethiopia, a study conducted in Babile district, Eastern Ethiopia, and a study conducted in Rawalpindi, India, which was 21.4%, 21.6%, 21.6%, and 20% [2, 19, 22, 37]. However, the prevalence in the current study was much lower than that of studies conducted in Eastern Tigray which was 33.7% [4], a study conducted in four zones (central, eastern, north-western, and southern zones) of Tigray, Northern Ethiopia, which was 58.3% [20], and studies conducted in different areas of India which was 25.7%, 28.2%, 43.5%, and 48.0%, respectively [13, 23, 34, 38, 39]. As thinness is an indicator of acute undernutrition, this difference might be explained in terms of the current improvements in nutrition, health, and socioeconomic status among study participants. The other justification may be the impact of the current commitment of the Ethiopian government for the improvement of nutritional conditions.

However, the above finding of this study contradicts with the study findings of Mekelle city, a study finding of Amhara Region, a study finding in Haryana district of India, and a study finding in Kathmandu, Nepal, which were 14%, 13.6%, 13%, and 9.5%, respectively [28, 35, 40, 41]. The variation in prevalence could be explained in socioeconomic and urban–rural difference between the study subjects and settings. In addition, as most of the thin girls had an attack of illness (diarrhea, malaria, and pneumonia). This might contribute to being thin as a recent episode of illness causing loss of weight and resulting in thinness, even though disease status has no statistically significant association in the current study.

When compared between younger/early and older/late adolescent girls, the prevalence of stunting was higher in older female adolescents than in younger ones (35.6% vs. 25.7%). This study identified that older adolescent girls were 2 times more likely to be stunted than their counterparts. The finding is consistent with other studies conducted in different areas such as in Garhwalbin, in Mieso, Somali region, in Kathmandu, and in Tamil Nadu, India, respectively [13, 24, 35, 39]. This might be due to poor/inadequate nutrient intake despite increased requirement during adolescent girls' faster growth period. Another possible explanation might be because this is a vulnerable period in girls as they will then be approaching the marriageable age and would be expected to be pregnant and deliver babies which could explain the fact that the prevalence of stunting increases as the age of the girls increases.

The study identified that those adolescent girls whose mothers work outside home were more likely to be stunted than those who do not work outside home or are housewives. Adolescent girls whose mothers' occupation is farmer and government employee were 2.4 times and 3 times more likely to be stunted than those adolescent girls whose mothers are housewives. This finding is in agreement with studies conducted in Kersa district of Eastern Ethiopia and in West Bengal, India [17, 42]. This might be explained by a reduced time of working mothers to care for their children at early childhood which in turn might affect the feeding practice that adversely affects the linear growth. Stunting that occurred during early childhood may progress into adolescent age.

This study also revealed that girls from educated mothers were less likely to be stunted than noneducated ones. Those adolescents whose mothers attended secondary and more than secondary education were 47.0% and 75.0% less likely to be stunted, respectively, as compared to girls whose mothers have no formal education. This finding goes in line with the study conducted in different parts of Ethiopia and other developing countries [14, 22, 36, 38, 42–44]. This is due to the fact that as the level of education of the mother increases, so do her finances as to increased productivity and her contribution to the total family income. This places the family at better nutritional status. In addition, education can also enable the women to make independent decisions that will improve nutrition and health of their children and to have greater access to household resources that are vital for nutritional status. Moreover, educated women are more aware of personal hygiene, promotive and curative health care, the type of food being prepared and distributed, and better methods of feeding than uneducated or less educated women in a particular family.

The result also showed that adolescent girls from poor families were 2 times more likely to be stunted than adolescent girls from rich one. This finding is similar with the study report in Mieso, Somali region, and with other studies [18, 24, 31, 32]. This may imply that adolescents' nutritional status is just dependent on the socioeconomic status of the households in which they reside. It causes inadequate quality and quantity of food intake due to inability to purchase variety and preferences of the type of food. It indicated that low socioeconomic status was found to be an important risk factor for stunting.

Regarding factors associated with thinness, current study identified that father's educational status was associated with thinness. Adolescent girls whose fathers had primary and secondary education level were 52.0% and 55.0% less likely to be thin than those from noneducated fathers. The finding goes in line with other studies from Adama and Mekelle and a study in Beni-Suef Governorate, Egypt [36, 40, 43]. This might explain that educational attainment of fathers could lead to a higher income which may imply higher availability of food and household resources. It might be positively associated with high nutritional awareness as well as better caring of their children and parents as a whole.

Similarly, adolescent girls whose mothers completed primary education were 44.0% less likely to be thin as compared to those who did not have formal education. This study is in agreement with the studies conducted in Adama and Adwa, Ethiopia, and Assam, India [22, 36, 38]. This might be because when the level of education of the mother is low, her decision making power and her contribution to the total family income will be low. This places the family at risk of not meeting their needs including nutritional needs. It may also affect the type of food prepared and distributed including the type of care received by the girls in a particular family.

This study also revealed that the number of meals being eaten per day was significantly associated with thinness. Adolescent girls who usually eat two and less meals per day were 1.8 times more likely to be thin as compared to those who usually eat three meals per day. The finding of this study is consistent with the studies done in Adwa, Northern Ethiopia, in Bedele town, southwestern Ethiopia, and in Bale zone, southeast Ethiopia [5, 22, 25]. This might be because skipping of meals leads to inadequate dietary intake. Adolescence period has the fastest growth and the nutritional requirements are increased to promote this growth spurt. Therefore, in addition to the increased nutritional demand during adolescence period, skipping of meals leads to being thin.

Although the study is first of its kind in the stated area, the authors acknowledge some limitations in this study. Some variables such as food security were not included. Data on behavioural and feeding habit may be affected by social desirability bias but during data collection adequate information was given to participants about the importance and confidentiality of information to minimize the bias. Other limitation of this study was that this study was conducted in rural areas and do not generalize to urban adolescent girls population. The measurement errors could be another possible limitation but standardization of measurements/instruments was done on daily basis.

Adolescence in girls is the most critical period but the adolescent girls are often thought of as healthy and strong and are often not considered within the development agenda as they are not children and not quite adults. According to current study, the prevalence of undernutrition, particularly that of stunting which is considered as index of chronic undernutrition, indicates that the poor nutritional status of girls remains uninterrupted throughout their adolescent life. Therefore, targeting adolescence can provide an opportunity to prevent the onset of nutrition related chronic diseases in adults' life and possibly also corrects some nutritional problems originating in the past. So, programs to support adequate nutrition for adolescents could provide an opportunity for healthy transition from childhood to adulthood and could be an important step towards breaking the vicious cycle of intergenerational malnutrition.

## 5. Conclusion

The prevalence of stunting and thinness among adolescent girls was moderate when compared to the prevalence reported in Sub-Saharan Africa. However, when the prevalence of both stunting and thinness was compared to the national nutrition baseline survey reports in Ethiopia, it was major public health problem. The likelihood of being stunted was significantly higher among older age adolescent girls, with lack of maternal education and in girls living in lower wealth status households. The likelihood of being thin was significantly higher among adolescent girls whose fathers and mothers lack formal education and those who eat meals only twice or less per day. Moreover, the likelihood of both being stunted and being thin was increasing as the age of adolescents increases.

Routine screening, assessment, counselling, and monitoring of adolescent girls' nutritional status at community level and implementing evidence based community nutrition programmes are important steps towards alleviating the problem. Educating parents of adolescent girls at all levels is a useful step in reducing the prevalence of stunting and thinness. Further research has to be conducted including adolescents from both urban and rural areas in addition to incorporating additional nutritional assessment methods.

## Figures and Tables

**Table 1:** Sociodemographic and economic characteristics among adolescent girls in Damot Sore Woreda, Southern Ethiopia, 2017.

Variables	Categories	Percentage
Girls' age (*n* = 719)	10–14 years	60.5
	15–19 years	39.5

Religion (*n* = 719)	Protestant	52.0
	Orthodox	24.7
	Apostolic	16.3
	Catholic	7.0

Ethnicity (*n* = 719)	Wolaita	96.5
	Others^a^	3.5

Family size (*n* = 719)	≤5	39.2
	>5	60.8

Father's occupation (*n* = 719)	Farmer	49.4
	Merchant	36.6
	Daily labourer	5.8
	Government employee	5.6
	Others^b^	2.6

Mother's occupation (*n* = 719)	House wife	44.0
	Merchant	39.8
	Farmer	9.2
	Government employee	4.5
	Others^c^	2.5

Father's education (*n* = 719)	No formal education	26.6
	Primary education	39.2
	Secondary education	26.0
	More than secondary education	8.2

Mother's education (*n* = 719)	No formal education	40.3
	Primary education	43.3
	Secondary education	11.5
	More than secondary education	4.9

Wealth index (*n* = 719)	Rich	36.2
	Medium	30.0
	Poor	33.8

Source/origin of food (*n* = 719)	Own product and market purchase	62.4
	Own product	28.5
	Market purchase	9.1

Frequency of meals per day (*n* = 719)	Two times	13.1
	Three times	62.6
	Four times	24.3

Regular meals skipped (*n* = 719)	No	97.5
	Yes	2.5

Episode of household hunger (*n* = 719)	No/little	98.5
	One time	1.5

Type of latrine (*n* = 719)	Pit latrine with slab	67.7
	Pit latrine without slab/open pit	32.3

Source of drinking water (*n* = 719)	Tap water	87.9
	Spring water	10.0
	Others^d^	2.1

Hand washing prior to meal (*n* = 719)	≤2 times	13.8
	3 times	76.7
	≥4 times	9.5

Soap using when washing hands (*n* = 719)	≤2 times	2.0
	3 times	68.8
	≥4 times	29.2

^a^Dawuro, Gamo, and Gofa. ^b^Pastor, student, and self-employee. ^c^Private employee and student. ^d^Protected well, nonprotected well, and river water.

**Table 2:** Factors associated with stunting during bivariable and multivariable analysis among adolescent girls in Damot Sore Woreda, Southern Ethiopia, 2017.

Variables	Stunting		COR (95% CI)	AOR (95% CI)
	Yes (*N*, %)	No (*N*, %)		
Girls' age				
10–14 years	112 (25.7)	323 (74.3)	1	1
15–19 years	101 (35.6)	183 (64.4)	1.60 (1.15–2.20)^*∗∗*^	2.06 (1.08–3.92)^*∗*^

Mother's age				
35–49 years	147 (28.8)	364 (71.2)	1	1
20–34 years	21 (23.3)	69 (76.7)	0.75 (0.45–1.27)	0.71 (0.40–1.24)
≥50 years	45 (38.1)	73 (61.9)	1.53 (1.01–2.32)^*∗*^	1.26 (0.80–1.98)

Family size				
≤5	91 (32.3)	191 (67.7)	1.23 (0.89–1.70)	1.30 (0.91–1.86)
>5	122 (27.9)	315 (72.1)	1	1

Father's occupation				
Farmer	93 (26.2)	262 (73.8)	1	1
Merchant	86 (32.7)	177 (67.3)	1.37 (0.96–1.94)	1.48 (0.99–2.20)
Daily labourer	12 (28.6)	30 (71.4)	1.13 (0.55–2.30)	1.05 (0.49–2.27)
Government employee	14 (35.0)	26 (65.0)	1.52 (0.76–3.03)	1.86 (0.60–5.79)
Others	8 (42.1)	11 (57.9)	2.05 (0.80–5.25)	1.62 (0.56–4.61)

Mother's occupation				
Housewife	90 (28.4)	227 (71.6)	1	1
Merchant	70 (24.5)	216 (75.5)	0.82 (0.57–1.18)	0.78 (0.53–1.17)
Farmer	30 (45.5)	36 (54.5)	2.10 (1.22–3.62)^*∗∗*^	2.38 (1.31–4.33)^*∗∗*^
Government employee	17 (53.1)	15 (46.9)	2.86 (1.37–5.97)^*∗∗*^	3.05 (1.35–6.92)^*∗∗*^
Others	6 (33.3)	12 (66.7)	1.26 (0.46–3.46)	1.42 (0.49–4.11)

Father's education				
No formal education	58 (30.4)	133 (69.6)	1	1
Primary education	69 (24.5)	213 (75.5)	0.74 (0.49–1.12)	0.64 (0.41–1.03)
Secondary education	68 (36.4)	119 (63.6)	1.31 (0.85–2.01)	1.19 (0.73–1.93)
More than secondary education	18 (30.5)	41 (69.5)	1.01 (0.53–1.89)	0.69 (0.24–1.98)

Mother's education				
No formal education	101 (34.8)	189 (65.2)	1	1
Primary education	83 (26.7)	228 (73.3)	0.68 (0.48–0.96)^*∗*^	0.69 (0.47–1.02)
Secondary education	23 (27.7)	60 (72.3)	0.72 (0.42–1.23)	0.53 (0.28–0.98)^*∗*^
More than secondary education	6 (17.1)	29 (82.9)	0.38 (0.16–0.96)^*∗*^	0.25 (0.09–0.69)^*∗∗*^

Number of meals per day				
Three times	130 (28.9)	320 (71.1)	1	1
≥four times	49 (28.0)	126 (72.0)	0.96 (0.65–1.41)	1.08 (0.70–1.67)
≤two times	34 (36.2)	60 (63.8)	1.39 (0.87–2.23)	1.52 (0.91–2.53)

Soap using when washing hands				
≤two times	6 (42.9)	8 (57.1)	2.28 (0.76–6.87)	2.47 (0.73–8.43)
Three times	155 (31.3)	340 (68.7)	1.38 (0.96–1.99)	1.36 (0.91–2.02)
≥ four times	52 (24.8)	158 (75.2)	1	1

Started menarche				
No	102 (27.1)	275 (72.9)	1	1
Yes	111 (32.5)	231 (67.5)	1.29 (0.94–1.78)	0.72 (0.38–1.35)

Wealth index				
Higher/rich	65 (25.0)	195 (75.0)	1	1
Medium	50 (23.1)	166 (76.9)	0.90 (0.59–1.38)	0.86 (0.55–1.35)
Lower/poor	98 (40.3)	145 (59.7)	2.03 (1.38–2.96)^*∗∗∗*^	1.94 (1.29–2.92)^*∗∗*^

^*∗*^ *p* value <0.05; ^*∗∗*^*p* value <0.01; ^*∗∗∗*^*p* value ≤0.001.

**Table 3:** Factors associated with thinness during bivariable and multivariable analysis among adolescent girls in Damot Sore Woreda, Southern Ethiopia, 2017.

Variables	Thinness		COR (95% CI)	AOR (95% CI)
	Yes (*N*, %)	No (*N*, %)		
Girls' age				
10–14 years	78 (17.9)	357 (82.1)	1	1
15–19 years	62 (21.8)	222 (78.2)	1.28 (0.88–1.86)	1.08 (0.56–2.07)

Family size				
≤5	64 (22.7)	218 (77.3)	1.39 (0.96–2.02)	1.38 (0.94–2.06)
>5	76 (17.4)	361 (82.6)	1	1

Father's education				
No formal education	55 (28.8)	136 (71.2)	1	1
Primary education	47 (16.7)	235 (83.3)	0.49 (0.32–0.77)^*∗∗*^	0.48 (0.31–0.77)^*∗∗*^
Secondary education	27 (14.4)	160 (85.6)	0.42 (0.25–0.69)^*∗∗*^	0.45 (0.26–0.78)^*∗∗*^
More than secondary	11 (18.6)	48 (81.4)	0.57 (0.27–1.17)	0.73 (0.33–1.60)

Mother's education				
No formal education	73 (25.2)	217 (74.8)	1	1
Primary education	46 (14.8)	265 (85.2)	0.52 (0.34–0.78)^*∗∗*^	0.56 (0.37–0.87)^*∗∗*^
Secondary education	13 (15.7)	70 (84.3)	0.55 (0.29–1.06)	0.58 (0.29–1.18)
More than secondary	8 (22.9)	27 (77.1)	0.88 (0.38–2.03)	0.85 (0.35–2.05)

Number of meals per day				
Three times	84 (18.7)	366 (81.3)	1	1
≥four times	25 (14.3)	150 (85.7)	0.73 (0.45–1.18)	0.72 (0.43–1.19)
≤two times	31 (33.0)	63 (67.0)	2.14 (1.31–3.50)^*∗∗*^	1.87 (1.12–3.13)^*∗*^

Soap using when washing hands				
≤two times	2 (14.3)	12 (85.7)	1.00 (0.21–4.69)	0.78 (0.15–3.99)
Three times	108 (21.8)	387 (78.2)	1.67 (1.07–2.60)^*∗*^	1.52 (0.95–2.41)
≥four times	30 (14.3)	180 (85.7)	1	1

Started menarche				
No	63 (16.7)	314 (83.3)	1	1
Yes	77 (22.5)	265 (77.5)	1.45 (0.99–2.09)	1.46 (0.77–2.77)

Wealth index				
Rich	46 (17.7)	214 (82.3)	1	1
Medium	37 (17.1)	179 (82.9)	0.96 (0.59–1.55)	0.92 (0.56–1.52)
Poor	57 (23.5)	186 (76.5)	1.43 (0.92–2.20)	1.25 (0.79–1.98)

^*∗*^ *p* < 0.05; ^*∗∗*^*p* < 0.01. COR = crude odds ratio and AOR = adjusted odds ratio.

## Data Availability

The datasets during and/or analysed during the current study are available from the corresponding author upon reasonable request.

## Abbreviations

AOR: Adjusted odds ratio
BMI: Body mass index
BAZ: Body mass index-for-age *z*-score
CDC: Center for Disease Prevention and Control
CHIS: Community health information system
CI: Confidence interval
COR: Crude odds ratio
HAZ: Height-for-age *z*-scores
OR: Odds ratio
PCA: Principal component analysis
SPSS: Statistical packages for social sciences
TEM: Technical error measurement
VIF: Variance inflation factor.
